# Sleep abnormalities are associated with greater cognitive deficits and disease activity in Huntington's disease: a 12-year polysomnographic study

**DOI:** 10.1093/braincomms/fcaf126

**Published:** 2025-04-02

**Authors:** Zanna J Voysey, Anna O G Goodman, Lorraine Rogers, Jonathan A Holbrook, Alpar S Lazar, Roger A Barker

**Affiliations:** Department of Clinical Neurosciences, John van Geest Centre for Brain Repair, University of Cambridge, Cambridge CB2 0PY, UK; Department of Clinical Neurosciences, John van Geest Centre for Brain Repair, University of Cambridge, Cambridge CB2 0PY, UK; Royal Papworth Hospital Foundation Trust, Sleep Centre, Cambridge CB2 0AY, UK; Department of Clinical Neurosciences, John van Geest Centre for Brain Repair, University of Cambridge, Cambridge CB2 0PY, UK; Faculty of Medicine and Health Sciences, University of East Anglia, Norwich NR4 7TQ, UK; Department of Clinical Neurosciences, John van Geest Centre for Brain Repair, University of Cambridge, Cambridge CB2 0PY, UK; Cambridge Stem Cell Institute, University of Cambridge, Cambridge CB2 0AW, UK

**Keywords:** polysomnography, actigraphy, neurodegeneration, dementia, neurofilament light

## Abstract

Increasing evidence suggests that the sleep pathology associated with neurodegenerative diseases can in turn exacerbate both the cognitive deficits and underlying pathobiology of these conditions. Treating sleep may therefore bear significant, even disease-modifying, potential for these conditions, but how best and when to do so remains undetermined. Huntington's disease, by virtue of being an autosomal dominant neurodegenerative disease presenting in mid-life, presents a key ‘model’ condition through which to advance this field. To date, however, there has been no clinical longitudinal study of sleep abnormalities in Huntington's disease and no robust interrogation of their association with disease onset, cognitive deficits and markers of disease activity. Here, we present the first such study. Huntington's disease gene carriers (*n* = 28) and age- and sex-matched controls (*n* = 21) were studied at baseline and 10- and 12-year follow-up. All Huntington's disease gene carriers were premanifest at baseline and were stratified at follow-up into ‘prodromal/manifest’ versus ‘premanifest’ groups. Objective sleep abnormalities were assessed through two-night inpatient polysomnography and 2-week domiciliary actigraphy, and their association was explored against Montreal Cognitive Assessment, Trail A/B task, Symbol Digit Modalities Task (SDMT), Hopkins Verbal Learning Task (HVLT) and Montgomery–Asberg Depression Rating Scale (MADRS) scores, plus serum neurofilament light levels. Statistical analysis incorporated cross-sectional ANOVA, longitudinal repeated measures linear models and regressions adjusted for multiple confounders including disease stage. Fifteen Huntington's disease gene carriers phenoconverted to prodromal/early manifest Huntington's disease by study completion. At follow-up, these gene carriers showed more frequent sleep stage changes (*P* ≤ 0.001, η_p_^2^ = 0.62) and higher levels of sleep maintenance insomnia (defined by wake after sleep onset, *P* = 0.002, η_p_^2^ = 0.52). The latter finding was corroborated by nocturnal motor activity patterns on follow-up actigraphy (*P* = 0.004, η_p_^2^ = 0.32). Greater sleep maintenance insomnia was associated with greater cognitive deficits (Trail A *P* ≤ 0.001, *R*^2^ = 0.78; SDMT *P* = 0.008, *R*^2^ = 0.63; Trail B *P* = 0.013, *R*^2^ = 0.60) and higher levels of neurofilament light (*P* = 0.015, *R*^2^ = 0.39). Longitudinal modelling suggested that sleep stage instability accrues from the early premanifest phase, whereas sleep maintenance insomnia emerges closer to phenoconversion. Baseline sleep stage instability was able to discriminate those who phenoconverted within the study period from those who remained premanifest (area under curve = 0.81, *P* = 0.024). These results demonstrate that the key sleep abnormalities of premanifest/early Huntington's disease are sleep stage instability and sleep maintenance insomnia and suggest that the former bears value in predicting disease onset, while the latter is associated with greater disease activity and cognitive deficits. Intervention studies to interrogate causation within this association could not only benefit patients with Huntington's disease but also help provide fundamental proof-of-concept findings for the wider sleep–neurodegeneration field.

## Teaser

Voysey *et al*. studied sleep in Huntington's disease gene carriers in a 12-year longitudinal study using polysomnography and actigraphy. They found that sleep abnormalities emerge many years prior to motor features, may form a predictive biomarker of disease onset and, importantly, are associated with greater cognitive deficits and disease activity.

## Graphical Abstract

**Graphical Abstract:**
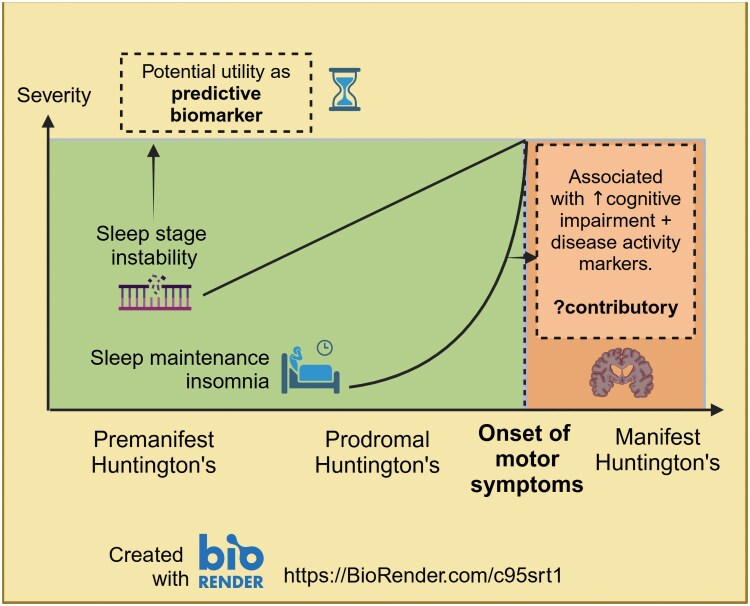

## Introduction

Both objective and subjective sleep abnormalities are highly prevalent across the spectrum of neurodegenerative disease,^1,2^ and growing evidence suggests a deleterious, feed-forward cycle in which the sleep disruption caused by neurodegeneration in turn exacerbates both the cognitive/affective features and pathophysiology of these conditions.^3-5^ Sleep disturbance is known, for example, to impair executive function,^6,7^ attention,^8^ processing speed^9,10^ and emotional regulation^11^ and also to promote neuroinflammation,^12^ aberrant protein homeostasis^13,14^ and impaired neuronal DNA repair.^15,16^ Further, sleep is purported to play a critical role in synaptic modulation supporting memory consolidation^17^ and in glymphatic clearance of neurotoxic species such as beta-amyloid and tau from the brain.^18,19^

Treating sleep disturbance therefore bears significant, even disease-modifying, potential for neurodegenerative conditions, and the recent emergence of new sleep therapies such as orexin antagonists^20^ makes this prospect all the more feasible.

Huntington's disease is a fully penetrant autosomal dominant neurodegenerative disease caused by a CAG repeat expansion mutation in the *huntingtin* gene. It is characterized by a combination of motor, cognitive and psychiatric features, with disease onset (‘manifest’ disease) defined by the development of unequivocal motor signs, typically occurring between the ages of 35 and 50.^21^

By virtue of these characteristics, Huntington’s disease enables sleep abnormalities to be studied longitudinally from prior to disease onset, facilitating fundamental insights into the ‘chicken’ and ‘egg’ of this feed-forward cycle. Further, it allows their relation to cognitive/affective features to be studied free from the confounding effects of advanced age or comorbid health conditions. This poses a key advantage over more common neurodegenerative conditions, which occur in late age and for which a pre-symptomatic or early phase can only be identified in retrospect. Alongside this, a number of preclinical transgenic animal model studies have suggested that treating sleep abnormalities can improve cognitive and survival outcomes in Huntington’s disease.^22-26^ Thus, Huntington’s disease poses a key ‘model’ condition through which to advance the sleep–neurodegeneration field.

The gold standard method for detecting objective sleep abnormalities is inpatient polysomnography (PSG), in which specialized EEG and EMG are recorded alongside measures of respiratory function and eye movements during sleep. This provides high-precision but short-duration sleep data recorded in an unnatural environment. Actigraphy, whereby participants wear a motion-sensitive device on a wrist for an extended period in the home environment, therefore provides a complementary objective measure to PSG, by providing lower-precision but long-duration data in an ecologically valid setting. Cross-sectional polysomnographic and actigraphic studies in manifest Huntington’s disease patients^27-33^ have suggested Huntington’s disease's main sleep abnormalities to comprise (i) low sleep efficiency due to high levels of wake after sleep onset (herein interpreted as sleep maintenance insomnia), (ii) sleep stage instability and (iii) increased light sleep with reduced slow wave sleep and rapid eye movement (REM) sleep. These features appear to become more prominent with disease progression.^29,32,33^ This profile mirrors that seen in Alzheimer's and Parkinson's diseases,^34-37^ supporting the potential validity of Huntington’s disease as a ‘model’ condition.

Nonetheless, many of these existing studies are limited in several ways, for example, a lack of habituation to PSG, a lack of definitive genetic diagnosis in early studies, heterogeneity of disease stage and failure to control for medication use or affective state. Furthermore, there has been only one study to date of sleep in premanifest Huntington’s disease gene carriers—a study by our group.^27^ This study suggested sleep stage instability and sleep maintenance insomnia to also occur at this disease stage, but without proportionate gain/loss of sleep stages.

To date, there has been no longitudinal study of sleep in Huntington’s disease patients and no robust interrogation of associations between sleep abnormalities and disease onset or clinical features. The handful of studies that have considered the latter are heavily limited either by the use of subjective measures of sleep,^38-40^ which are known to be unreliable in Huntington’s disease,^28^ or by failure to control for age.^41,42^

Huntington’s disease also has the advantage that there is an easily obtainable putative biomarker of disease activity in its prodromal/early stages, namely, serum neurofilament light (NfL). A number of studies^43-47^ have recently demonstrated that NfL exhibits a sigmoidal trajectory in Huntington’s disease, with rapid increases during the late premanifest/transitional phase. Yet to date, there has also been no study exploring the relationship between objective sleep abnormalities and NfL in Huntington’s disease.

Here, we aimed to address these knowledge gaps through an exploratory study. We studied a cohort of Huntington’s disease gene carriers and age- and sex-matched healthy controls at three time points over a 12-year period. All Huntington’s disease gene carriers were premanifest at baseline; approximately half had converted to prodromal/early manifest Huntington’s disease by study completion. Sleep abnormalities were assessed by both inpatient PSG and domiciliary actigraphy, and their relationship was explored versus both cognitive/affective features and NfL levels.

Results are intended to inform the design of targeted sleep intervention trials in Huntington’s disease. Such studies bear potential not only to bring benefit to Huntington’s disease patients but also to answer fundamental proof-of-concept questions regarding the contribution of sleep abnormalities to the presentation and progression of neurodegeneration.

## Materials and methods

### Study design

**Figure 1:**
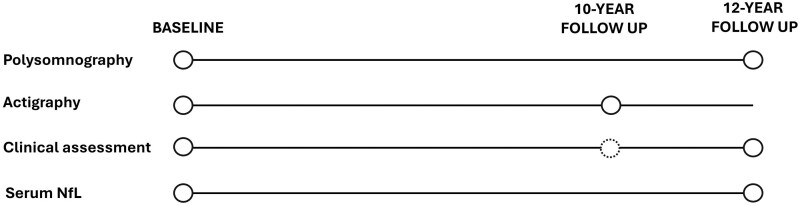
**Study design.** The dashed circle denotes where assessments were undertaken by video call in place of in-person assessment and where UHDRS TMS examination was precluded, due to Covid-19 restrictions preventing face-to-face assessments. NfL, neurofilament light.

Study structure, including timing of the study subcomponents, is depicted in Fig. 1. This structure reflected the influence of Covid-19 restrictions, which precluded face-to-face assessments at the time of 10-year follow-up. At each time point, all subcomponents were undertaken within a maximum of 12 months of one another.

### Recruitment

Twenty-eight patients and 22 controls were recruited in 2009–10. Approximately 50% of Huntington’s disease gene carriers were recruited from the Cambridge HD Clinic; the remainder self-referred from other Huntington’s disease clinics across the UK. Approximately 50% of controls constituted healthy partners of recruited gene carriers; the remainder were recruited by local advertisement. Ethical approval was granted by local ethics committees, and all participants provided written informed consent in accordance with the Declaration of Helsinki (REC 03/187 and 15/EE/0445).

Baseline inclusion criteria comprised (i) a positive genetic test for Huntington’s disease, conferred by CAG repeat length ≥ 38 (in Huntington’s disease gene carriers) and (ii) a Unified Huntington's Disease Rating Scale Diagnostic Confidence Level (UHDRS DCL) of 0–1 (in Huntington’s disease gene carriers). The latter equates to <50% clinician confidence of signs of Huntington’s disease, ensuring that all Huntington’s disease gene carriers were premanifest at baseline. Baseline exclusion criteria comprised (i) diagnosis of a sleep disorder (in controls), (ii) diagnosis of any other neurodegenerative/neuroinflammatory condition or traumatic brain injury and (iii) diagnosis of a psychiatric condition bar mild–moderate anxiety or depression.

**Figure 2:**
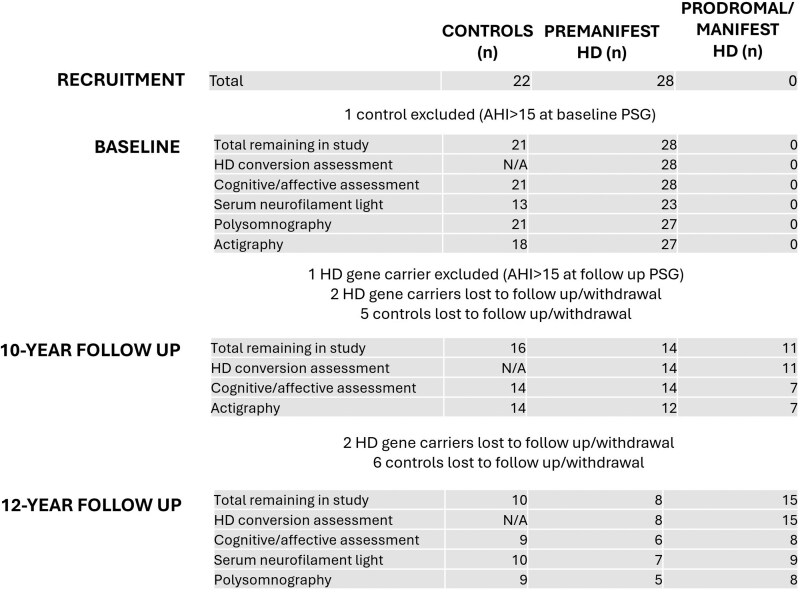
**Participation and conversion flow chart:** totals across study time points. AHI, apnoea–hypopnea index, N/A, non-applicable.

Participants were subsequently also excluded from analysis if there was (i) evidence of untreated moderate sleep apnoea during PSG, defined as apnoea–hypopnoea index (AHI) > 15^48^ (*n* = 2; Fig. 2), (ii) diagnosis of any other neurodegenerative/neuroinflammatory condition or traumatic brain injury during the follow-up period (*n* = 0), (iii) diagnosis of a psychiatric condition bar mild–moderate anxiety or depression during the follow-up period (*n* = 0) or (iv) night shift work or travel > 2 time zones from UK < 2 weeks prior to study assessments (*n* = 0).

### Clinical assessment

The following standardized, validated clinical assessments were selected for their established sensitivity both to features of prodromal and early Huntington’s disease^49^ and to the effects of sleep disturbance.^6,9-11^

- Montreal Cognitive Assessment: test of global cognition, score 0–30, higher score equates to better performance.
- Trail Making Test part A (Trail A): test of attention and psychomotor speed, timed 0–180 s, shorter time equates to better performance.
- Trail Making Test part B (Trail B): test of executive function, timed 0–180 s, shorter time equates to better performance.
- Hopkins Verbal Learning Test-Revised, delayed recall component (HVLT delayed): test of learning and memory, score 0–12, higher score equates to better performance.
- Symbol Digit Modalities Task (SDMT): test of attention and psychomotor speed, score 0–110, higher score equates to better performance.
- Montgomery–Asberg Depression Rating Scale (MADRS): clinician rated measure of depression, range 0–60, score 7–19 = mild depression, 20–34 = moderate depression.

At follow-up, Huntington’s disease gene carriers were assessed for evidence of conversion to prodromal or manifest Huntington’s disease according to their UHDRS total motor score (UHDRS TMS, score 0–124) and UHDRS DCL (range 0–4, score ≥ 2 indicative of >50% confidence of signs of manifest Huntington’s disease). UHDRS TMS scores were restricted to 12-year follow-up due to Covid-19 restrictions preventing face-to-face examinations at 10-year follow-up. Prodromal and manifest individuals were considered as one group; individuals who remained premanifest were considered as a separate group. Groups were defined according to the following criteria:

- Premanifest: UHDRS DCL 0–1, plus UHDRS TMS 0–3 where UHDRS TMS score was available (i.e. at 12-year follow-up)
- Prodromal/manifest: UHDRS DCL ≥ 2, plus UHDRS TMS ≥ 4 where UHDRS TMS score was available (i.e. at 12-year follow-up)

These thresholds were selected on the basis of those recommended and used within major consensus papers within Huntington’s disease literature.^50-52^ The more recent HD Integrated Staging System (HD-ISS) categories^53^ could not be employed as an alternative due to the absence of MRI brain imaging within our data set.

Conversion status was also incorporated where it could be ascertained from routine clinical care records and/or study participation before or after study time points. For example, where participants remained premanifest at 12-year follow-up, it could be reliably inferred that they had been premanifest at 10-year follow-up, and conversely where participants reached the above criteria for prodromal/manifest Huntington’s disease during clinical care assessments prior to 10-year follow-up, prodromal/manifest status could be reliably inferred at 10-year follow-up.

UHDRS total functional capacity scoring (UHDRS TFC, range 0–13) was also undertaken at baseline and 12-year follow-up to provide a measure of disease severity (11–13 = early stage; 7–10 = early–mid stage Huntington’s disease). All UHDRS components were undertaken by certified clinicians, and all clinical assessments were undertaken blinded to sleep assessment results.

In order to mitigate against the potential effects of confounders, data were also collected during clinical assessment regarding a number of demographic factors (Table 1). Predicted years to onset of manifest Huntington’s disease at baseline was calculated using the Langbehn formula at 60% probability,^54^ a formula based on age and CAG repeat length. Precise baseline–follow-up interval was determined by months between clinical assessments. Other confounding factor variables were recorded as binary yes/no data. History of relevant medical comorbidity was defined as those reported by participants to disrupt to sleep on average ≥ 2 times per week, including perimenopausal symptoms. Use of relevant medications was defined as those for which drowsiness, sleep disorder, confusion, impaired concentration or impaired memory was listed as a common side effect in the British National Formulary 2023. History of alcohol excess was defined as >14units/week for women and >21units/week for men, for ≥3-month period. History of caffeine excess was defined as >400 mg/day for ≥3-month period. For participants undertaking actigraphy, i.e. a domiciliary metric, the presence of a cohabitant causing regular sleep disruption (average ≥ 2 times/week) was also recorded.

**Table 1:** Baseline cohort demographics

Demographic variable	Controls	Huntington’s disease	*P*
*n*	21	28	N/A
Age (mean, SD)	45.4 (16.5)	44.0 (11.4)	NS
Sex (% male)	42.9	32.1	NS
CAG repeat length (mean, SD)	N/A	41.1 (2.0)	N/A
Predicted years to onset from baseline (mean, SD)	N/A	18.0 (10.1)	N/A
Years of education (mean, SD)	15.4 (3.6)	14.7 (2.4)	NS
In employment (%)	88.2	83.3	NS
Relevant medications (%) a	14.3	25	NS
History of alcohol excess (%)	25	34.8	NS
History of caffeine excess (%)	12.5	43.5	NS
Relevant medical comorbidities (%) b	0	8.3	NS
History of anxiety/depression (%)	38.9	46.2	NS
Cohabitant causing sleep disruption (%)	0	0	NS

Group differences assessed by independent Student's *t*-test or *χ*^2^/Fisher's exact test.

N/A, non-applicable; NS, non-significant.

^a^Relevant medications for Huntington’s disease gene carriers: carbamazepine (*n* = 1), SSRI (*n* = 5), SNRI (*n* = 1), benzodiazepine (*n* = 1), beta-blocker (*n* = 1), statin (*n* = 1). Relevant medications for controls: SSRI (*n* = 1), ACE inhibitor (*n* = 1), calcium channel blocker (*n* = 1).

^b^Relevant medical comorbidities for Huntington’s disease gene carriers: post nasal drip (*n* = 1) and fibromyalgia (*n* = 1). Where % and *n* are incongruent, this reflects isolated cases of missing data, polypharmacy and/or multiple comorbidities.

### Neurofilament light: meso scale discovery assay

Due to the known effect of advanced age^55^ and renal impairment^56^ on serum NfL levels, participants were excluded from the NfL subcomponent where baseline age was >65 (*n* = 3) or where there was a diagnosis of renal impairment (*n* = 0). Extracted serum from venous blood samples was stored at −80°C until processing. NfL concentrations were determined using the Meso Scale Discovery S-PLEX Neurology Panel 1 (Human) kit according to the manufacturer's instructions and methodology described in our parallel publication.^47^ All values fell within the dynamic range of the assay (1.7–1400 pg/mL).

### Polysomnography

PSG was undertaken via an inpatient study over two consecutive nights (first night habituation, second night used for data analysis) in a light, temperature and noise-controlled laboratory environment. Participants were asked to follow their typical sleep/wake routine and to refrain from caffeine intake or naps during their inpatient stay. A full clinical PSG setup was used in 10–20 EEG distribution (Fz, Cz, Pz, F3/4, C3/4, P3/4, T3/4, O1/O2, A1/A2), including electrooculography, tibialis anterior and submental electromyography surface electrodes. Respiratory function was assessed via pulse oximetry, nasal cannulae with pressure transducer and thoracic respiratory effort belt. PSG signals were recorded using an Embla S7000 and visualized using RemLogic software (Embla Systems, Ontario, Canada). EEG was recorded with reference electrodes at mastoid areas (A1 and A2) with a common reference electrode at Pz. EEG signals were stored at 200 Hz, with a low-pass filter at 70 Hz and high-pass filter at 0.3 Hz.

Sleep staging was performed in 30 s epochs according to Rechtschaffen and Kales criteria (R&K) by scorers blinded to participant identity and disease group. R&K were used as American Academy of Sleep Medicine (AASM) criteria were not established at the time of the baseline time point; their continued use at follow-up was therefore required to facilitate comparison between time points, as well as between our current data set and that derived from the same cohort in previous publications.^27^ AHI and periodic limb movements were scored according to standard criteria.^48,57^ Scoring of follow-up PSG was cross-checked by the same individuals who had scored the baseline time point PSG, in order to mitigate against potential inter-rater variability.

Multiple objective PSG variables were calculated, based on standard objective sleep features used in clinical research, with the addition of three variables at follow-up (Table 2). The latter reflected expanded variables of interest identified in emergent literature during the study period.^27,30^ Non-proportional PSG variables occurring within sleep (arousals, sleep stage changes and limb movements) were normalized to total sleep time; those relating to wakefulness during the sleep period (awakenings and wake after sleep onset) were normalized to total time in bed. PSG data were excluded where total sleep time was <3 h, if this was <50% of the minimum total sleep time recorded during the participant's corresponding 14 nights of actigraphy (*n* = 1).

**Table 2:** PSG outcomes

PSG variables	Baseline			12-year FU				
	Controls	Huntington’s disease	*Q*	Controls	Premanifest Huntington’s disease	Prodromal/Manifest Huntington’s disease	*Q*	η_p_^2^
*n*	21	27	N/A	9	5	8	N/A	.
TIB (hours)	8.1 (1.0)	8.6 (0.8)	NS	8.8 (0.8)	8.4 (1.6)	9.6 (1.0)	NS	.
TST (hours)	6.9 (1.0)	7.2 (1.2)	NS	8.1 (0.8)	8.0 (1.3)	7.8 (1.3)	NS	.
Sleep efficiency (%)	85.3 (7.8)	84.1 (9.8)	NS	92.0 (3.2)***	94.9 (3.5)**	80.6 (10.8)	**0.001**	**0.56**
Sleep onset latency (min)	15.5 (17.8)	17.4 (15.3)	NS	8.0 (2.9)	5.4 (3.0)	12.5 (16.2)	NS	.
Stage 1 (%)	12.0 (5.4)	10.7 (4.6)	NS	8.9 (3.5)	11.3 (5.3)	12.6 (5.2)	NS	.
Stage 2 (%)	51.7 (5.9)	51.7 (8.5)	NS	43.3 (3.5)	41.5 (6.9)	37.8 (7.1)	NS	.
Slow wave sleep (%)	17.0 (7.2)	17.4 (8.1)	NS	23.1 (5.2)	19.1 (4.4)	28.3 (6.6)	NS	.
REM sleep (%)	19.3 (6.0)	21.0 (5.0)	NS	24.7 (5.8)	31.3 (3.3)**	20.9 (5.2)	**0.03**	.
Wake after sleep onset (min/hour)	6.8 (3.8)	6.4 (3.8)	NS	3.9 (2.0)**	2.4 (2.0)***	10.3 (5.6)	**0.005**	**0.52**
Awakenings/hour	1.2 (0.7)	1.3 (0.7)	NS	1.6 (0.7)	1.2 (0.4)	2.2 (1.1)	NS	.
Limb movement arousals/hour	2.8 (1.1)	3.4 (1.4)	NS	5.7 (3.3)	5.7 (1.7)	5.9 (2.9)	NS	.
Sleep stage changes/hour	25.6 (6.7)	26.2 (8.0)	NS	25.1 (6.9)**	23.6 (3.8)***	34.0 (6.1)	**0.001**	**0.62**
Limb movements/hour a	.	.	.	9.7 (5.2)	11.2 (2.0)	9.0 (4.5)	NS	.
Periodic limb movements/hour a	.	.	.	3.2 (3.8)	1.7 (2.2)	1.5 (1.9)	NS	.
Arousals/hour a	.	.	.	13.0 (3.9)	12.6 (4.8)	18.6 (4.3)	NS	.

Group differences assessed by ANCOVA adjusted for age, sex, CAG repeat length, MADRS depression score, relevant medication use and individual interval between baseline and follow-up. Values represent totals, % or mean (SD) as applicable. *P < 0.05, **P < 0.01, ***P < 0.001 in *post hoc* Tukey test versus prodromal/manifest Huntington’s disease group.

N/A, non-applicable; NS, non-significant; FU, follow-up; TIB, time in bed; TST, total sleep time; *Q*, *P* value with false discovery rate correction applied.

^a^Variable added to analysis protocol at 12-year follow-up. Effect size reported where *P* < 0.05 in both pairwise *post hoc* assessments versus prodromal/manifest Huntington’s disease. Note that direct comparison cannot be made between results at different time points, due to participant attrition between time points; please see longitudinal modelling results for this analysis.

### Actigraphy

Actigraphy was undertaken via 14 consecutive day/night domiciliary recordings during which participants wore an actiwatch (MotionWatch8, CamNTech, Cambridge, UK) continuously on their non-dominant wrist. Participants were instructed to follow their habitual sleep/wake patterns and to complete a daily sleep diary to document recording anomalies (for example, non-representative rest-activity (RA) patterns due to transient intercurrent illness, or where there was delay in re-siting an actiwatch following bathing): such data periods were excluded (Table 3).

**Table 3:** Actigraphy outcomes

Actigraphy variables	Baseline			10-year FU				
	Controls	Huntington’s disease	*Q*	Controls	Premanifest Huntington’s disease	Prodromal/manifest Huntington’s disease	*Q*	η_p_^2^
*n*	18	27	N/A	14	12	7	N/A	.
Days of actigraphy	13.7 (0.6)	13.9 (0.6)	NS	13.9 (0.4)	13.8 (0.5)	13.7 (0.4)	NS	.
L5 activity count	1160 (592)	1405 (662)	NS	1266 (615)**	1209 (472)**	2494 (1027)	**0.028**	**0.32**
M10 activity count	25 084 (5671)	26 438 (7128)	NS	33 368 (10 423)	31 988 (6664)	40 788 (12 386)	NS	.
L5 onset (48 h clock time)	25.1 (0.7)	25.0 (1.2)	NS	25.1 (0.7)	24.7 (1.3)	24.7 (2.0)	NS	.
M10 onset (48 h clock time)	33.2 (1.5)	33.3 (1.6)	NS	32.5 (0.7)	32.5 (1.4)	32.3 (1.5)	NS	.
Relative amplitude	0.92 (0.04)	0.89 (0.10)	NS	0.92 (0.04)	0.92 (0.03)	0.87 (0.08)	NS	.
Interdaily stability	0.54 (0.13)	0.52 (0.11)	NS	0.51 (0.16)	0.58 (0.08)	0.60 (0.11)	NS	.
Intradaily variability	0.77 (0.20)	0.72 (0.17)	NS	0.88 (0.28)	0.79 (0.13)	0.70 (0.15)	NS	.

Group differences assessed by univariate general linear models adjusted for age, sex, CAG repeat length, MADRS depression score, relevant medication use and individual interval between baseline and follow-up. Values represent totals or mean (SD) as applicable. *P < 0.05, **P < 0.01, ***P < 0.001 in *post hoc* Tukey test versus prodromal/manifest Huntington’s disease group. Effect size reported where *P* < 0.05 in both pairwise *post hoc* assessments versus prodromal/manifest Huntington’s disease.

N/A, non-applicable; NS, non-significant; FU, follow-up; Q, *P* value following false discovery rate correction.

Actiwatches comprised triaxial accelerometers recording peak intensity of movement each second, sampled at 50 Hz, expressed as an activity count in 30 s epochs. Actograms were analysed using MotionWare software (CamNTech, Cambridge, UK) according to previously published non-parametric circadian rhythm analysis algorithms.^28^ In brief, the time of onset and mean activity levels during the lowest 5 h of activity (i.e. reflecting nocturnal sleep, L5), and highest 10 h of activity (M10), are calculated for each 24 h period and averaged over the recording period. L5 and M10 are then combined to generate a measure of the relative amplitude of RA levels. Interdaily stability is calculated to provide an indication of regularity of RA patterns across days (scale 0–1, higher indicating greater stability), while intradaily variability is calculated to provide an indication of consolidation of rest versus activity within days (scale 0–2, values > 1 indicating abnormal fragmentation). By adopting this approach, actigraphy data did not require adjustment according to participant-estimated bed/wake times. Since such estimates are liable to inaccuracy/interindividual variability in precision, this maximized the reliability of results.

### Statistical analysis

Statistical analysis was undertaken in SPSS v29.0.0 (IBM, Armonk, NY, USA). The threshold for statistical significance for all analyses was *P* < 0.05 (two-tailed).

Outlier data points were defined as those falling > 3 SDs from group means and causing skew from normal distribution and were excluded. This applied to <0.5% (*n* = 8) data points across the entire data set.

Cross-sectional group differences were assessed by one-way analysis of covariance (ANCOVA).

Longitudinal group differences were assessed by repeated measures general linear models. Where significant group*time interactions were identified, additional corroboratory evidence from linear mixed models was sought, incorporating group, time and covariates as fixed effects and individual participant intercepts as random effects. This was implemented due to the presence of loss to follow within the data set: this approach therefore reduced the potential for false positive results caused by the data loss incumbent in a repeated measures design based on a data set with an, while also avoiding sole reliance on estimated data in a mixed model design.

Associations between sleep variables and clinical outcomes/NfL levels were assessed by multivariate linear regression.

Variables included within and/or residuals resulting from parametric models were assessed for normal distribution by Shapiro–Wilk test and QQ plot, with variables transformed where necessary.

Covariate adjustment was based on both the a priori strength of effect of known biological confounders (e.g. age, sex, depression and relevant medication use) and the presence/absence of group differences in confounders within our data set. Given (i) the high number of potential confounders relevant to sleep and cognitive data, (ii) the necessarily low number of observations given the rarity of the condition and extended time period of follow-up and (iii) the likelihood of combinatorial effects and collinearity between relevant covariates, we judged combined backward and forward selection of covariates, alongside assessment for collinearity by variance inflation factor, to represent the most parsimonious and stringent approach to our data set.^58^ Specifically, within ANCOVAs and longitudinal linear models, we adjusted for age, sex, CAG repeat length, MADRS depression score, relevant medication use and baseline–follow-up interval time via stepwise backward elimination, with age and sex then resubmitted (where previously eliminated) by forward selection. This approach was then modified for regression analyses between sleep metrics and clinical measures among Huntington’s disease gene carriers, since the baseline–follow-up interval was no longer relevant, but adjustment for disease stage became important. In these analyses, we therefore replaced baseline–follow-up interval with predicted years to disease onset at baseline. Since the latter is derived from a formula based on CAG repeat length,^54^ CAG repeat length was omitted from the stepwise protocol to avoid collinearity.

Group differences in PSG and actigraphy metrics were adjusted for multiple comparisons by Benjamini–Hochberg correction^59^ (false discovery rate < 0.05), given the high number of derived variables.

Effect sizes for group differences were expressed as partial eta square (η_p_^2^) or Cohen's *f*^2^, as applicable.

Receiver operating characteristic (ROC) curve and area under the curve (AUC) analyses were used to explore the ability of sleep variables to discriminate phenoconversion patterns among Huntington’s disease gene carriers over the study period.

## Results

### Demographics

The baseline cohort comprised 21 controls (43% male, age 45.4 ± 16.5) and 28 premanifest Huntington’s disease gene carriers (32% male, age 44.0 ± 11.4). Huntington’s disease gene carriers were on average 18.0 ± 10.1 years from predicted conversion to manifest Huntington’s disease at baseline, with a CAG range of 38–46 (Table 1). No participant had a diagnosed sleep disorder at baseline. The distribution between controls, premanifest Huntington’s disease and prodromal/manifest Huntington’s disease gene carriers was 21:28:0 at baseline, 16:14:11 at 10-year follow-up and 10:8:15 at 12-year follow-up. Figure 2 details precise rates of participation in each study subcomponent and cases of exclusion and loss to follow-up/withdrawal.

Huntington’s disease gene carriers and controls did not differ with respect to confounding factors at baseline (Table 1). Similarly, groups did not differ in any of these factors at 10-year and 12-year follow-up, other than with respect to precise baseline–follow-up interval, and, as would be expected, higher CAG repeats and lower predicted years to onset from baseline among Huntington’s disease gene carriers who converted to prodromal/manifest Huntington’s disease (Supplementary Tables 1–4). These factors were therefore included in covariate adjustment (see Materials and methods). There was also no evidence of significant withdrawal bias in gene carriers across the study period (Supplementary Tables 5–8).

### Clinical assessment

At baseline, Huntington’s disease gene carriers exhibited no differences from controls with respect to cognitive/affective measures (Supplementary Table 9). However, by 10-year follow-up, Huntington’s disease gene carriers who had converted to prodromal/manifest disease exhibited impaired attention and psychomotor speed (Trail A *F*(2,30) = 4.78, *P* = 0.016, η_p_^2^ = 0.24; SDMT *F*(2,30) = 16.39, *P* = <0.001, η_p_^2^ = 0.52), executive function (Trail B *F*(2,31) = 16.64, *P* = 0.002, η_p_^2^ = 0.52) and learning/memory (HVLT delayed *F*(2,27) = 19.15, *P* = <0.001, η_p_^2^ = 0.59) compared both to controls and Huntington’s disease gene carriers who had remained premanifest (Supplementary Table 9). These deficits were also evident in group differences at 12-year follow-up, with the exception of learning/memory (HVLT delayed) which no longer met statistical significance.

MADRS depression scores did not differ between groups at any time point, and only one participant met criteria for moderate depression at any follow-up time point (Huntington’s disease gene carrier; score = 25 at 12-year follow-up).

UHDRS TFC among prodromal/manifest Huntington’s disease gene carriers at 12-year follow-up was 10.9 ± 2.7, indicating that these participants remained in the early stages of disease by study completion (Supplementary Table 9).

To explore the possible influence of video versus in-person clinical assessment (due to Covid-19 restrictions), we compared results at 10- and 12-year follow-up in participants who had undergone both forms of assessment (*n* = 20). There were no significant differences in any clinical assessment result (Supplementary Table 10). To consider the possible impact of the absence of UHDRS TMS score in conversion assessments at 10-year follow-up (again due to Covid-19 restrictions), we also analysed discrepancies between 10- and 12-year follow-up group classifications. In no instance was a gene carrier scored as premanifest at 10-year follow-up but manifest at 12-year follow-up.

### Neurofilament light

At baseline, NfL concentrations did not differ significantly between Huntington’s disease gene carriers and controls, but were elevated among prodromal/manifest Huntington’s disease gene carriers at 12-year follow-up compared to both controls and gene carriers who had remained premanifest (Supplementary Table 9; *F*(2,23) = 14.7, *P* = 0.003, η_p_^2^ = 0.51). NfL levels between controls and premanifest Huntington’s disease gene carriers at 12-year follow-up were not significantly different (*F*(1,15) = 0.58, *P* = 0.88).

In summary, clinical assessment revealed no differences between Huntington’s disease gene carriers and controls at baseline, whereas at follow-up, Huntington’s disease gene carriers who had converted to prodromal/manifest disease had developed deficits in attention, psychomotor speed and executive function and higher NfL levels compared to both controls and gene carriers who remained premanifest. The clinical and NfL profiles seen in our study were therefore concordant with published epidemiological data in Huntington’s disease patients^49,60^ other than a relatively low prevalence of depression.^61^

### Polysomnography

**Figure 3:**
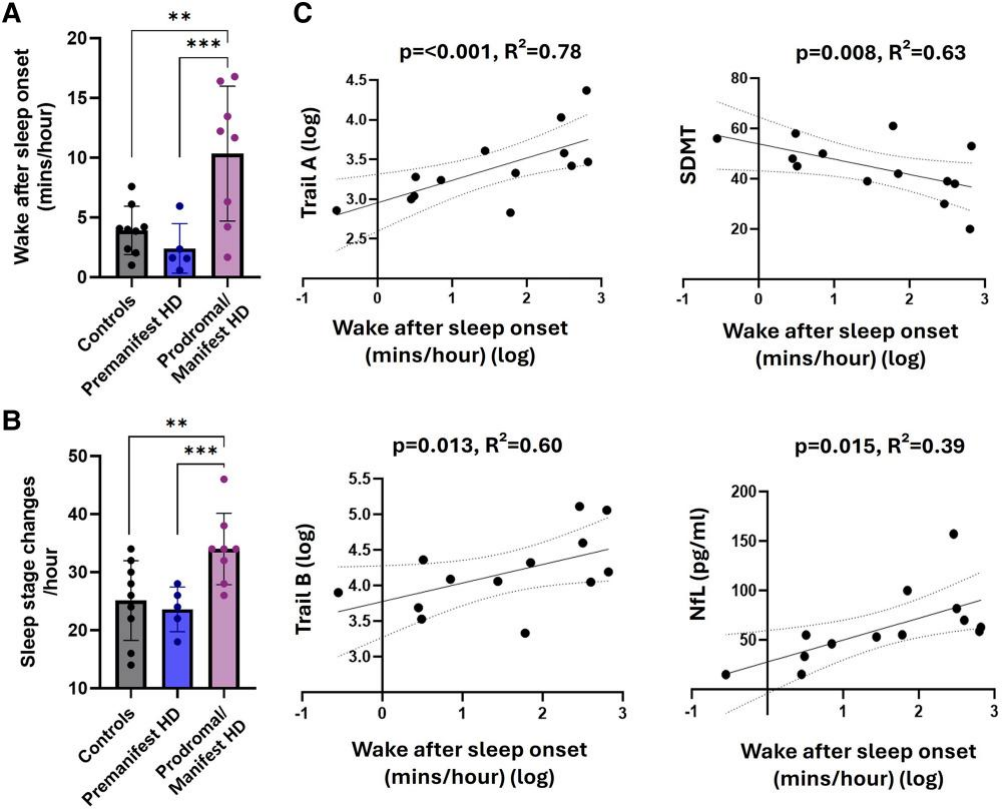
**PSG outcomes and associations with clinical markers.** Group differences in wake after sleep onset (**A**) and sleep stage changes (**B**) on PSG at 12-year follow-up, assessed by ANCOVA adjusted for age, sex, CAG repeat length, MADRS depression score, relevant medication use and individual interval between baseline and follow-up. Error bars = ± 1 SD. *P < 0.05, **P < 0.01, ***P < 0.001 in *post hoc* Tukey test. Individual data points represent data from individual participants and therefore indicate sample size. (**C**) Association between wake after sleep onset and clinical measures in Huntington’s disease gene carriers at 12-year follow-up, assessed by linear regression adjusted for age, sex, predicted years to onset at baseline, MADRS depression score and relevant medication use. Individual data points represent data from individual participants and therefore indicate sample size.

There were no differences in PSG variables between Huntington’s disease gene carriers and controls at baseline (Table 2). However, at 12-year follow-up, Huntington’s disease gene carriers who had converted to prodromal/manifest disease exhibited a number of abnormalities compared to both controls and premanifest Huntington’s disease gene carriers: cross-sectional analysis revealed a higher frequency of sleep stage changes (SSC *F*(2,17) = 13.65, *P* = <0.001, η_p_^2^ = 0.62), lower sleep efficiency (SE *F*(2,18) = 11.47, *P* = <0.001, η_p_^2^ = 0.56) and greater levels of wake after sleep onset (WASO *F*(2,18) = 9.62, *P* = 0.002, η_p_^2^ = 0.52) (Table 2 and Fig. 3). Correlation analysis demonstrated that the decline in sleep efficiency in these individuals was due to elevated WASO (*P* ≤ 0.001, ρ = −0.97) rather than sleep onset latency (SOL *P* = 0.25, ρ = −0.35). Total WASO indicated that 88% of prodromal/manifest Huntington’s disease gene carriers met clinical thresholds for sleep maintenance insomnia (WASO > 30 min^62^), compared to only 20% of premanifest Huntington’s disease gene carriers.

Supplementary Fig. 1 provides illustrative examples of PSG hypnograms from the three groups.

Longitudinal modelling via repeated measures ANCOVA revealed a significant group*time interaction for WASO (*F*(2,18) = 5.17, *P* = 0.017, η_p_^2^ = 0.37). This interaction also remained statistically significant following analysis via linear mixed model (*P* = 0.002, Cohen f^2^ = 0.53).

**Figure 4:**
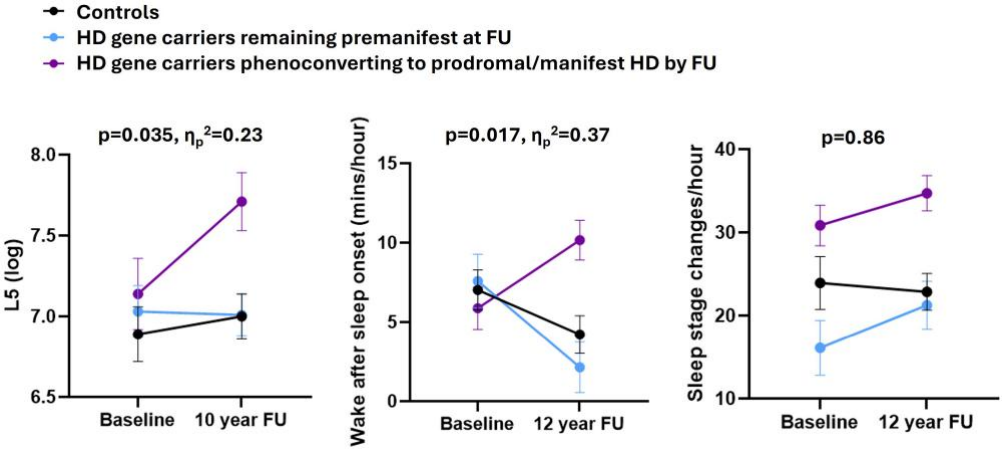
**Longitudinal modelling of actigraphy L5 (left), PSG wake after sleep onset (middle) and PSG sleep stage changes (right).** Values represent estimated marginal means ± 1 SEM. *P* values represent group*time interaction significance in repeated measures general linear models adjusted for age, sex, CAG repeat length, MADRS depression score, relevant medication use and individual interval between baseline and follow-up. FU, follow-up. Sample size distribution (controls:premanifest at FU:prodromal/manifest at FU) was left: *n* = 11:12:6; middle: *n* = 9:5:8; right *n* = 7:4:7.

By contrast, there was no such significant group*time interaction in a repeated measures ANCOVA of SSC: there were similar patterns of increase in SSC among gene carriers across the study period, irrespective of whether they remained premanifest or converted to prodromal/manifest Huntington’s disease by study completion, compared to stable levels among controls (Fig. 4; *F*(2,15) = 0.87, *P* = 0.86).

We then assessed for associations between these sleep abnormalities at 12-year follow-up and cognitive/affective features or NfL levels in Huntington’s disease gene carriers. We found no such association with respect to SSC. However, greater WASO was associated with higher NfL levels (*P* = 0.015, *R*^2^ = 0.39) as well as greater deficits in attention and psychomotor speed (Trail A *P* ≤ 0.001, *R*^2^ = 0.78; SDMT *P* = 0.008, *R*^2^ = 0.63) and executive function (Trail B *P* = 0.013, *R*^2^ = 0.60) at 12-year follow-up (Fig. 3). This association survived adjustment for multiple confounders including disease stage, relevant medication use and depression scores (Fig. 3). There was no association between WASO and MADRS depression scores (*P* = 0.59) or learning/memory (HVLT delayed, *P* = 0.14).

In summary, PSG revealed no differences between Huntington’s disease gene carriers and controls at baseline, whereas at follow-up, Huntington’s disease gene carriers who had converted to prodromal/manifest disease exhibited poorly consolidated sleep, characterized by high levels of WASO (i.e. sleep maintenance insomnia) and sleep stage instability. Greater sleep maintenance insomnia was in turn associated with higher NfL levels and greater deficits in attention, psychomotor speed and executive function. Longitudinal modelling suggested that sleep stage instability accrues at a similar rate during both early and late premanifest Huntington’s disease, whereas sleep maintenance insomnia emerges during the approach to conversion to manifest disease.

### Actigraphy

**Figure 5:**
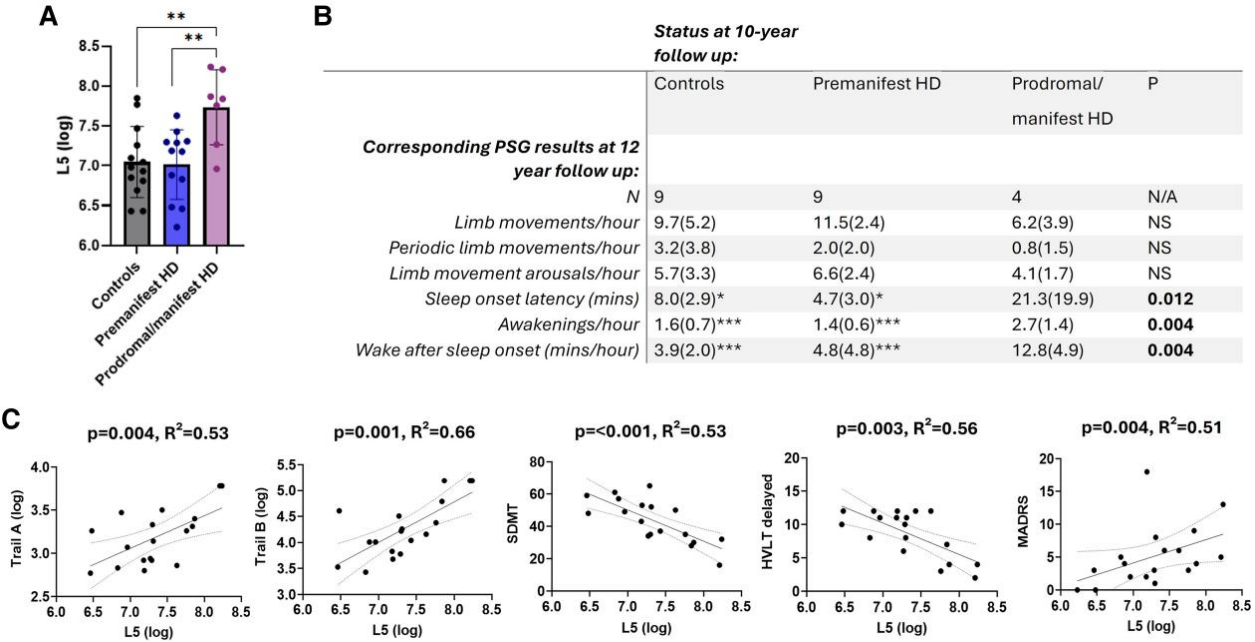
**Actigraphy outcomes and associations with clinical markers.** (**A**) Group differences in sleep period activity (L5) on actigraphy at 10-year follow-up, assessed by ANCOVA adjusted for age, sex, CAG repeat length, depression score, relevant medication use and individual interval between baseline and follow-up. Error bars = ± 1 SD. Individual data points represent data from individual participants and therefore indicate sample size. (**B**) Conversion status at 10-year follow-up in relation to relevant PSG variables at 12-year follow-up. *P < 0.05, **P < 0.01, ***P < 0.001 in *post hoc* Tukey test versus prodromal/manifest Huntington’s disease group. (**C**) Association between L5 and clinical measures in Huntington’s disease gene carriers at 10-year follow-up, assessed by linear regression adjusted for age, sex, predicted years to onset at baseline, MADRS depression score and relevant medication use. Individual data points represent data from individual participants and therefore indicate sample size.

At baseline, Huntington’s disease gene carriers did not differ from controls with respect to any actigraphy variable (Table 3). However, at 10-year follow-up, gene carriers who had converted to prodromal/manifest disease exhibited elevated levels of nocturnal motor activity (L5) compared to both premanifest gene carriers and controls (Table 3 and Fig. 5; *F*(2,29) = 6.75, *P* = 0.004, η_p_^2^ = 0.32). This was also reflected longitudinally, with a significant group*time interaction evident for L5 (Fig. 4; *F*(2,26) = 3.83, *P* = 0.035, η_p_^2^ = 0.23).

By chance, all Huntington’s disease gene carriers who had converted to prodromal or manifest Huntington’s disease at 10-year follow-up were female (Supplementary Table 2). Therefore, to further assess the possible influence of sex on our results beyond covariate adjustment, we also conducted female-only analysis across groups, in which the difference in L5 between groups at 10-year follow-up remained statistically significant (*F*(2,17) = 4.90, *P* = 0.021, η_p_^2^ = 0.37).

Since actigraphy purely reports movement, this finding could have reflected motor activity in sleep or motor activity during nocturnal wakeful periods. To interrogate this, we cross-referenced Huntington’s disease gene carriers’ actigraphy results at 10 years with their PSG results at 12 years. This revealed that individuals with high nocturnal movement on actigraphy (i.e. those who were prodromal/manifest during 10-year actigraphy) exhibited higher rates of wakefulness on their subsequent PSG (SOL *F*(2,19) = 5.60, *P* = 0.012,η_p_^2^ = 0.37; awakenings *F*(2,18) = 7.30, *P* = 0.004,η_p_^2^ = 0.44; WASO *F*(2,19) = 7.70, *P* = 0.004, η_p_^2^ = 0.45), but showed no difference with respect to movements within sleep (Fig. 5). Thus, the elevated nocturnal movement (L5) result more likely represented motor activity during periods of nocturnal wakefulness than movements in sleep. As groups did not differ with respect to daytime activity levels (M10; Table 3), it is unlikely that chorea during nocturnal wakefulness made a substantial contribution to this nocturnal motor activity.

We then assessed for associations between L5 and cognitive/affective features among Huntington’s disease gene carriers at 10-year follow-up. Higher L5 was associated with greater deficits in attention and psychomotor speed (Trail A *P* = 0.004, *R*^2^ = 0.53; SDMT *P* = <0.001, *R*^2^ = 0.53) as well as executive function (Trail B *P* = 0.001, *R*^2^ = 0.66) (Fig. 5). In addition, higher L5 was also associated with greater deficits in learning/memory (HVLT delayed *P* = 0.003, *R*^2^ = 0.56) and higher depression scores (MADRS *P* = 0.004, *R*^2^ = 0.51) (Fig. 5). These associations between L5 and cognitive deficits survived adjustment for disease stage, relevant medication use, depression scores and multiple other confounders (see Materials and methods).

In summary, actigraphy revealed no differences between Huntington’s disease gene carriers and controls at baseline, whereas at follow-up, Huntington’s disease gene carriers who had converted to prodromal/early manifest Huntington’s disease demonstrated higher levels of nocturnal wakefulness, i.e. sleep maintenance insomnia. Greater sleep maintenance insomnia was in turn associated with greater deficits in attention, psychomotor speed, executive function and memory and with greater depression. Thus, actigraphy data corroborated the findings from PSG data, with the addition of associations with memory and depression.

### Retrospective baseline analysis

We then explored whether sleep profiles varied by proximity to disease onset, or bore predictive value in estimating this. To achieve this, we retrospectively stratified the baseline cohort of Huntington’s disease gene carriers according to clinical outcome at study completion: those who had converted to prodromal/manifest disease by study completion were termed <12 years from conversion at baseline, whereas those who remained premanifest were termed >12 years from conversion at baseline.

When considering baseline sleep variables for these groups, Huntington’s disease gene carriers < 12 years from conversion exhibited elevated SSC compared to those > 12 years from conversion (mean 29.2 ± 8.5 versus 20.7 ± 5.8 changes/hour, *F*(1,17) = 12.32, *P* = 0.003, η_p_^2^ = 0.42). No other sleep variable, including WASO and L5, exhibited group differences. Thus, our results suggest that sleep stage instability develops earlier than sleep maintenance insomnia in premanifest Huntington’s disease gene carriers.

In support of this, ROC curve analysis revealed that baseline SSC showed good ability to discriminate Huntington’s disease gene carriers who phenoconverted to prodromal/manifest disease during the study period from those who remained premanifest (AUC = 0.81, *P* = 0.024), with a SSC cut off score of 23.2 changes/hour exhibiting 69% sensitivity and 71% specificity in determining this. Baseline WASO, by contrast, did not exhibit parallel efficacy (AUC = 0.51, *P* = 0.942) (Supplementary Fig. 2).

In summary, when Huntington’s disease gene carriers were stratified according to proximity to manifest disease, one difference in baseline sleep metric data emerged. Namely, sleep stage instability was higher in those < 12 years from manifest disease onset. In turn, baseline sleep stage instability exhibited predictive utility in identifying Huntington’s disease gene carriers at risk of phenoconversion within the study period.

## Discussion

Here, we present the first clinical study to examine the longitudinal dynamics of sleep abnormalities in Huntington’s disease and the first robust interrogation of the associations between these sleep abnormalities versus disease onset, clinical features and markers of disease activity.

Using objective PSG and actigraphy, we show that, although total sleep time and sleep stage proportions are preserved, patients with prodromal/early manifest Huntington’s disease exhibit less consolidated sleep, characterized by high levels of wake after sleep onset (sleep maintenance insomnia) and frequent sleep stage changes. Sleep stage instability appears to accrue gradually from early within the premanifest phase, whereas sleep maintenance insomnia appears later in this transition to manifest disease.

We then show, for the first time, that the more Huntington’s disease patients experience sleep maintenance insomnia, the worse their cognitive impairment (attention, psychomotor speed and executive function) and the higher their markers of disease activity (NfL). These associations survived adjustment for multiple confounders including disease stage, relevant medication use and depression scores. Clearly, causation cannot be inferred from these associations; they may represent epiphenomena. However, if it were the case that sleep maintenance insomnia was making a superadded contribution to these measures, it raises the possibility that treating this insomnia in prodromal/early Huntington’s disease patients could improve cognitive outcomes and/or disease progression. This would be significant, given that we currently have no proven therapies that improve cognition or survival in Huntington’s disease.

This hypothesis is supported by the number of preclinical studies that have demonstrated that treating sleep abnormalities in Huntington’s disease can improve cognitive and survival outcomes.^22-26^ Alongside this, an increasing number of studies in Alzheimer's and Parkinson's disease, in both animal models and humans, also suggest that enhancing sleep can positively modulate neurodegenerative pathophysiology.^63-67^ Moreover, many of the aforementioned postulated mechanisms by which sleep disturbance may drive neurodegeneration are highly relevant to Huntington’s disease. For example, neuroinflammation^68^ and altered proteostasis^69^ are recognized as key components driving Huntington’s disease pathophysiology, and likewise, CAG mutation somatic expansion due to impaired DNA repair is one of the current key focuses of the Huntington’s disease field.^70^ Likewise, recent evidence has suggested that mutant huntingtin is cleared by the glymphatic pathway,^71^ in addition to tau—which is also implicated in Huntington’s disease pathophysiology.^72^ Considering this evidence as a whole, therefore, it would appear reasonable to hypothesize that treating sleep abnormalities could mitigate the pathobiology of Huntington’s disease.

We also show, for the first time, that sleep features may carry value in predicting proximity to manifest Huntington’s disease, since levels of sleep stage instability were able to discriminate gene carriers who phenoconverted during the subsequent 12 years from those who did not. Importantly, this mirrors findings in more common neurodegenerative diseases, where sleep abnormalities including sleep fragmentation constitute a key component of the disease prodrome.^73,74^

Our identified sleep phenotype is consistent with our previous cross-sectional study.^27^ Nonetheless, it should be acknowledged that there was overlap of participants between these two studies: independent replication studies are therefore needed.

Our findings of a relationship between sleep maintenance insomnia and impaired attention and psychomotor speed and executive function is in line with evidence from healthy individuals.^6-10^ This is particularly the case given that sleep continuity has been found to bear greater influence on cognitive performance than total sleep time.^9,75^ To date, there have been few studies assessing these associations in the setting of neurodegeneration, but the limited evidence available would also suggest their presence in Alzheimer's and Parkinson's.^4,76,77^ Likewise, studies in Alzheimer's and Parkinson's have found correlation between sleep disturbance and NfL levels.^78,79^

Our study is notable for a number of strengths. The 12-year timespan of the study is, for example, not only the longest study of this type in Huntington’s disease but also enabled us to perform group stratification based on actual rather than predicted conversion outcomes. This is an advantage over the majority of Huntington’s disease studies in premanifest gene carriers, which base stratification on predicted proximity to disease onset; a prediction known to be of limited accuracy.^80^ Moreover, we controlled for a large number of potential confounding factors, many of which have been overlooked in previous studies of sleep in neurodegenerative conditions. Above all, it is striking that our PSG and actigraphy results provided independent corroboration of one another.

Nonetheless, our study has a number of limitations. Firstly, while our cohort was sizeable given the rarity of Huntington’s disease as a condition and the timeframe of follow-up, absolute numbers of participants undertaking some study subcomponents, particularly at follow-up PSG, were small. This sample size also meant that some confounders such as relevant medications had to be considered as binary yes/no variables, which clearly limits the precision to which they could be controlled for.

Linked to this, it is striking that we found an association between sleep maintenance insomnia (as indicated by L5 on actigraphy) and poorer memory performance and higher depression scores in Huntington’s disease gene carriers at 10-year follow-up, but did not find this relationship at 12-year follow-up (when considering sleep maintenance insomnia indicated by WASO). Indeed, the memory deficits that were evident in prodromal/manifest participants at 10-year follow-up were no longer evident at 12-year follow-up. Given that we found no evidence of withdrawal bias to account for this (Supplementary Table 8), this is likely to be due to the reduced cohort size at 12-year follow-up, which may have rendered us underpowered to detect some differences and relationships. The contribution of practice effects between 10- and 12-year follow-ups also cannot be entirely ruled out, given that alternate versions of HVLT were not used, and practice effects have been seen at a 1-year retest interval in Huntington’s disease patients.^81^ Likewise, the low rates of depression within our cohort as a whole may have limited our ability to detect relationships between depression and sleep abnormalities and may have been influenced by our entry exclusion criterion regarding severe anxiety/depression. However, this profile within our cohort argues against our findings being attributable to depression.

Secondly, the lack of a study time point midway through our study period also limits the precision with which we can estimate the longitudinal dynamics of Huntington’s disease sleep abnormalities. For example, we cannot infer at what point in the 12 years prior to conversion to prodromal/manifest disease Huntington’s disease gene carriers typically develop sleep maintenance insomnia—i.e. whether this typically occurs in the decade prior to, or more concomitantly with, motor manifestation of Huntington’s disease. Future studies should consider this.

Thirdly, as no Huntington’s disease gene carriers reached advanced disease by study completion, our findings cannot be extrapolated to later disease stages. Indeed, cross-sectional studies predict that the profile of sleep abnormalities evolves across the natural history of Huntington’s disease, with loss of REM and slow wave sleep emerging in moderate/advanced disease.^27,30,33^ This mirrors the phenotype seen in Alzheimer's and Parkinson's disease, where sleep fragmentation and insomnia form features of the disease prodrome, whereas loss of REM and slow wave sleep occur predominantly in established disease.^34,73,74,82,83^

Fourthly, the reduction in WASO between baseline and follow-up among controls and premanifest Huntington’s disease gene carriers suggests that participants may not have been fully habituated to PSG at baseline. Moreover, our interpretation of WASO as equivalent to sleep maintenance insomnia also carries an important caveat, as the clinical diagnosis of such insomnia requires associated daytime dysfunction, e.g. cognitive impairment, mood disturbance or fatigue. Since Huntington’s disease itself already causes such features, it is challenging to ascertain on an individual basis whether clinical diagnostic criteria for sleep maintenance insomnia are met.

Moreover, NfL remains an exploratory biomarker of disease activity in Huntington’s disease, and future studies would be enhanced by the incorporation of additional measures such as caudate thinning on volumetric MRI.^84^ Inclusion of MRI would also enable participants to be stratified according to the recent HD-ISS criteria.^53^

Above all, this study is limited as causation clearly cannot be inferred from the observed associations. Intervention studies are therefore now warranted to probe whether treating sleep maintenance insomnia in prodromal/early Huntington’s disease can improve cognition and/or disease progression. Such a study would not only benefit Huntington’s disease patients, but is also ideally placed to provide fundamental proof-of-concept findings regarding the contribution of sleep abnormalities to the features and pathobiology of neurodegeneration.

## Supplementary Material

fcaf126_Supplementary_Data

## Data Availability

The data that support the findings of this study are available on request from the corresponding author. The data are not publicly available due to their containing information that could compromise the privacy of research participants.
