# Supplementary material for: Sleep abnormalities are associated with greater cognitive deficits and disease activity in Huntington's disease: a 12-year polysomnographic study
Source: Brain Commun. 2025 Apr 2;7(2):fcaf126. doi: 10.1093/braincomms/fcaf126 (PMC11992570; doi:10.1093/braincomms/fcaf126)
Supplement: fcaf126_Supplementary_Data [file fcaf126_supplementary_data.docx]

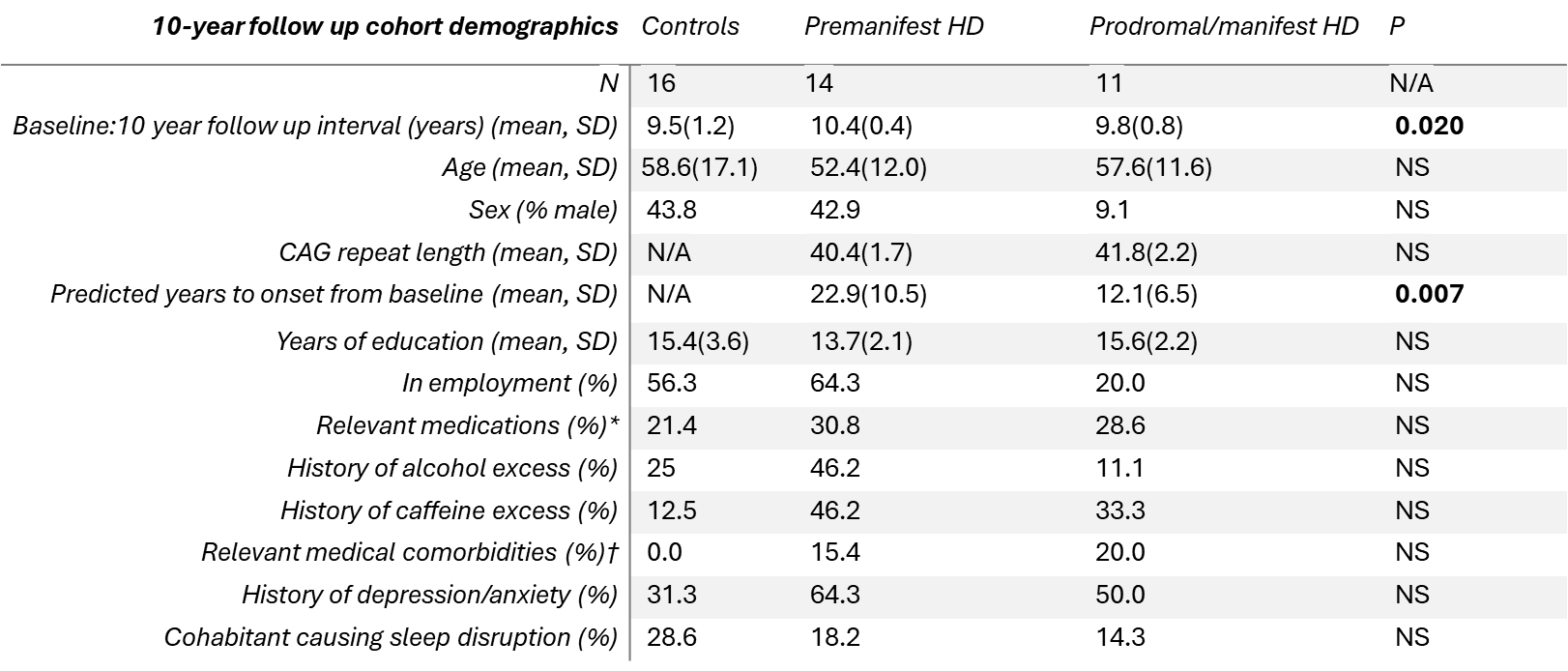


**Supplementary Table 1. Cohort demographics at 10-year follow up.** N/A=non-applicable. NS=non-significant. Group differences assessed by independent Student’s T-test, One-way ANOVA or Chi square/Fisher’s exact test. *Relevant medications in controls: beta blocker(n=1), statin(n=2), calcium channel blocker(n=1), ACE inhibitor(n=1). Relevant medications in HD gene carriers: carbamazepine(n=1), SSRI (n=2), statin(n=1), antihistamine(n=1), beta blocker (n=1), amantadine(n=1). †Relevant comorbidities in HD gene carriers: post nasal drip(n=1), fibromyalgia(n=1), perimenopausal symptoms(n=2). Where % and n are incongruent, this reflects isolated cases of missing data polypharmacy, and/or multiple comorbidities.

***
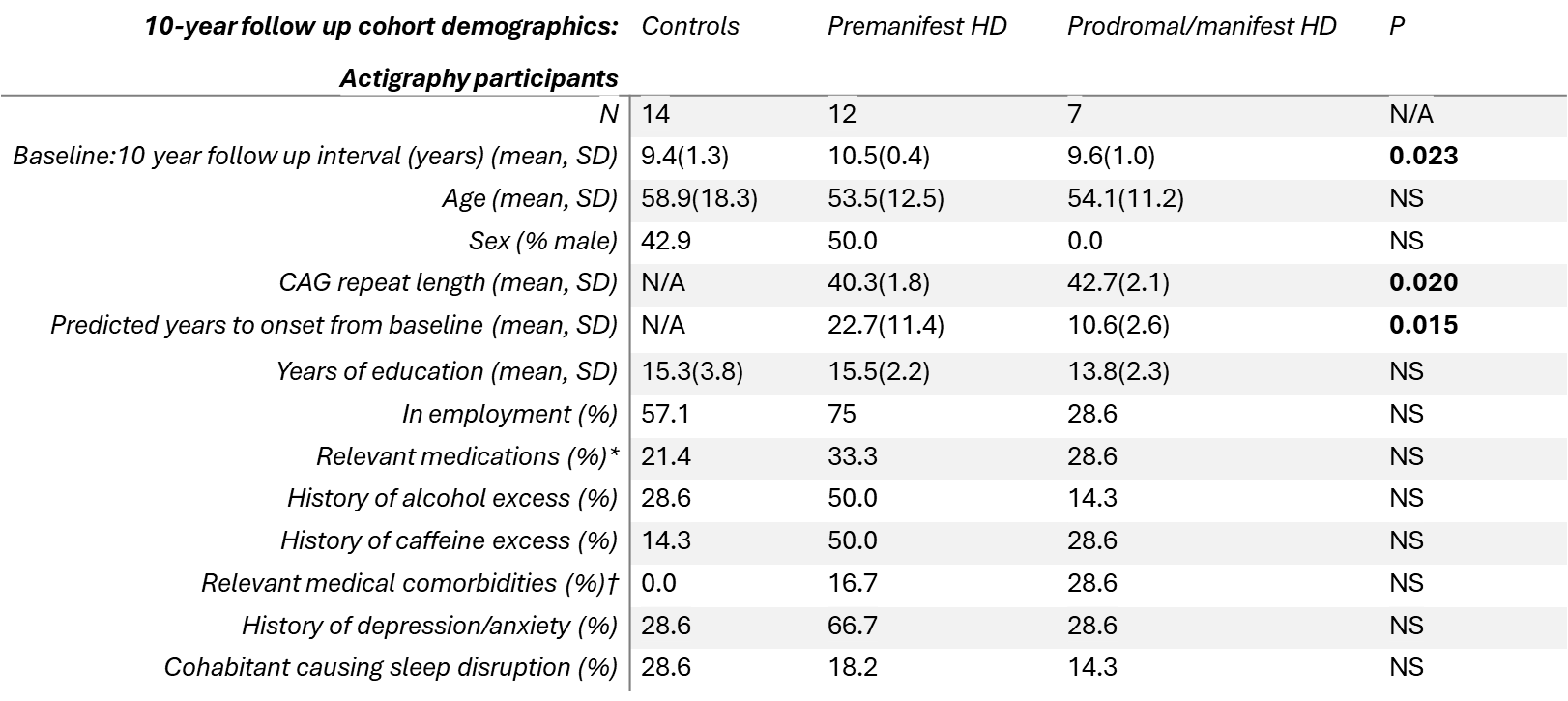
***

**Supplementary Table 2. Cohort demographics at 10-year follow up, limited to participants who undertook actigraphy.** N/A=non-applicable. NS=non-significant. Group differences assessed by independent Student’s T-test, One-way ANOVA or Chi square/Fisher’s exact test. *Relevant medications in controls: beta blocker(n=1), statin(n=2), calcium channel blocker(n=1), ACE inhibitor(n=1). Relevant medications in HD gene carriers: carbamazepine(n=1), SSRI (n=2), statin(n=1), antihistamine(n=1), beta blocker (n=1), amantadine(n=1). †Relevant comorbidities in HD gene carriers: post nasal drip(n=1), fibromyalgia(n=1), perimenopausal symptoms(n=2). Where % and n are incongruent, this reflects isolated cases of missing data, polypharmacy and/or multiple comorbidities.

***
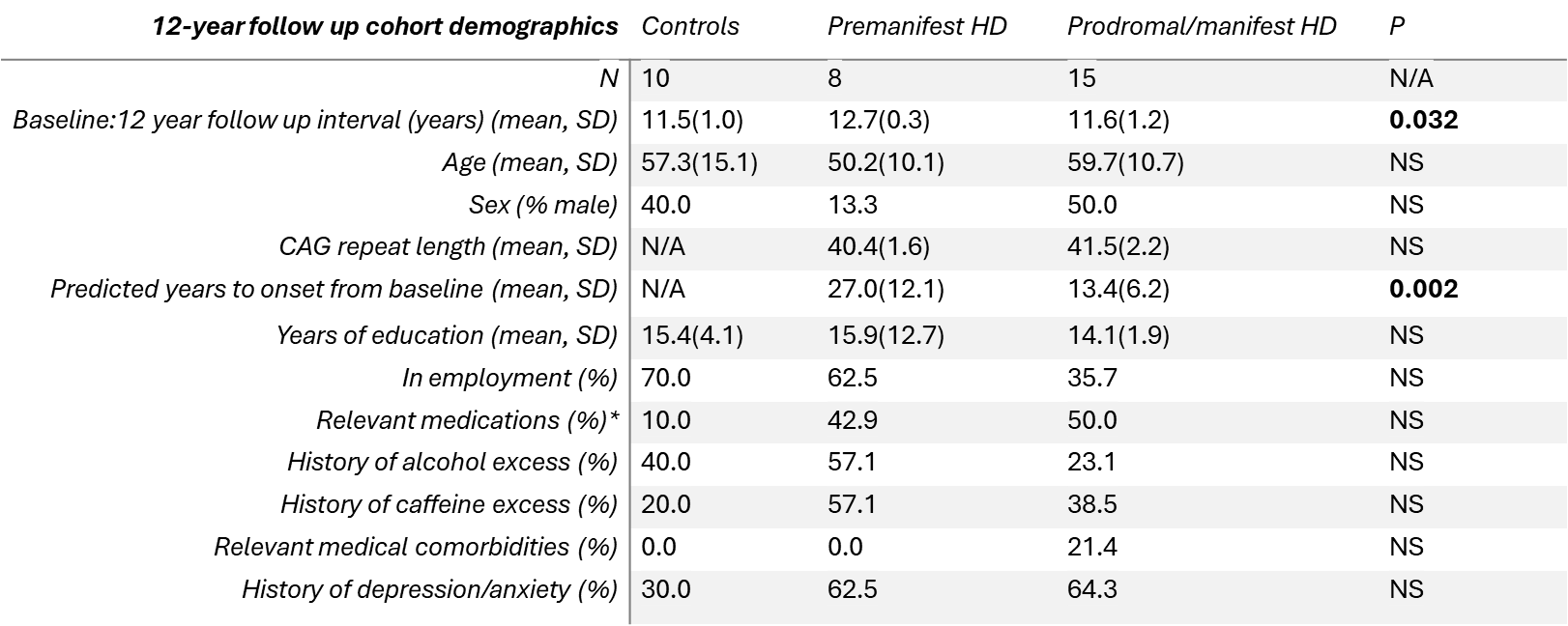
***

**Supplementary Table 3. Cohort demographics at 12-year follow up.** N/A=non-applicable. NS=non-significant. Group differences assessed by independent Student’s T-test, One-way ANOVA or Chi square/Fisher’s exact test. *Relevant medications in controls: statin(n=1), calcium channel blocker(n=1), ACE inhibitor(n=1). Relevant medications in premanifest HD: SSRI (n=4), SNRI(n=1), antihistamine(n=1), carbamazepine(n=1), beta blocker(n=1), statin(n=2), ACE inhibitor(n=1), rivastigmine(n=1). Where % and n are incongruent, this reflects isolated cases of missing data, polypharmacy and/or multiple comorbidities. Cohabitant status not recorded as no domestic recordings (actigraphy) undertaken at this timepoint.

***
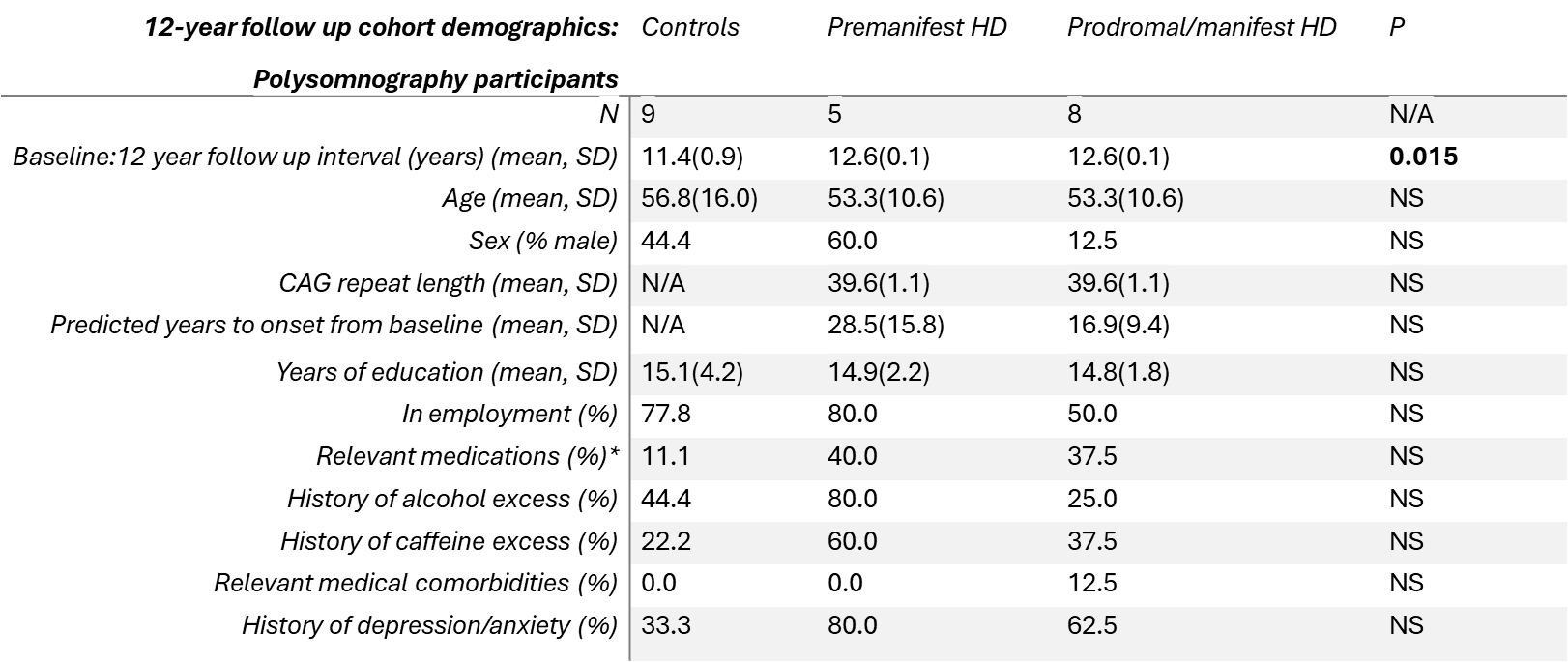
***

**Supplementary Table 4. Cohort demographics at 12-year follow up, limited to participants undertaking polysomnography.** N/A=non-applicable. NS=non-significant. Group differences assessed by independent Student’s T-test, One-way ANOVA or Chi square/Fisher’s exact test. *Relevant medications in controls: statin(n=1), calcium channel blocker(n=1), ACE inhibitor(n=1), beta blocker(n=1). Relevant medications in HD gene carriers: SSRI (n=3), carbamazepine(n=1), statin(n=1), rivastigmine(n=1). Where % and n are incongruent, this reflects isolated cases of missing data, polypharmacy and/or multiple comorbidities. Cohabitant status not recorded as no domestic recordings (actigraphy) undertaken at this timepoint.

***
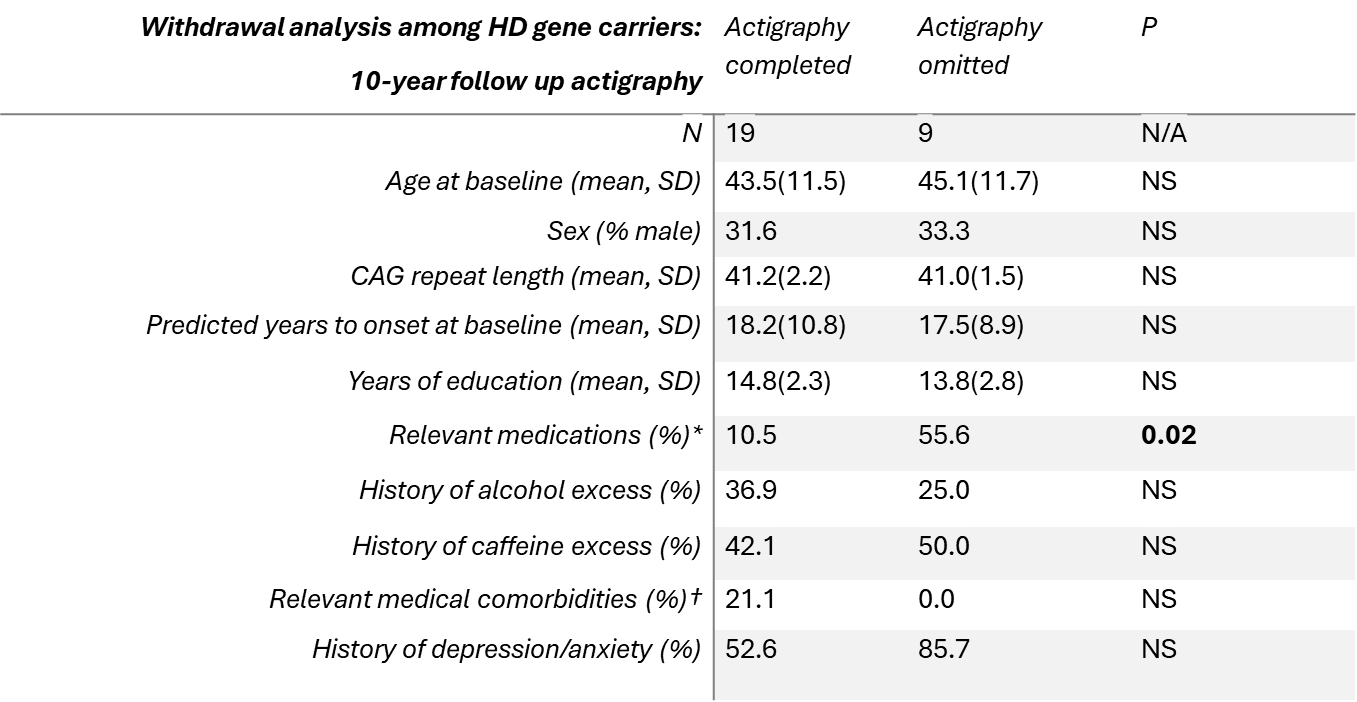
***

**Supplementary Table 5. Withdrawal bias analysis among HD gene carriers: actigraphy at 10-year follow up.** N/A=non-applicable. NS=non-significant. Group differences assessed by independent Student’s T-test or Chi square/Fisher’s exact test. Relevant medications in those completing actigraphy: carbamazepine(n=1), SSRI(n=1). Relevant medications in those omitting actigraphy: SSRI(n=4), SNRI(n=1), benzodiazepine(n=1), beta blocker(n=1), statin(n=1), ACE inhibitor(n=1), calcium channel blocker(n=1). Relevant comorbidities in those completing actigraphy: perimenopausal symptoms(n=2), post nasal drip(n=1), fibromyalgia(n=1). Where % and n are incongruent, this reflects isolated cases of missing data, polypharmacy and/or multiple comorbidities.

***
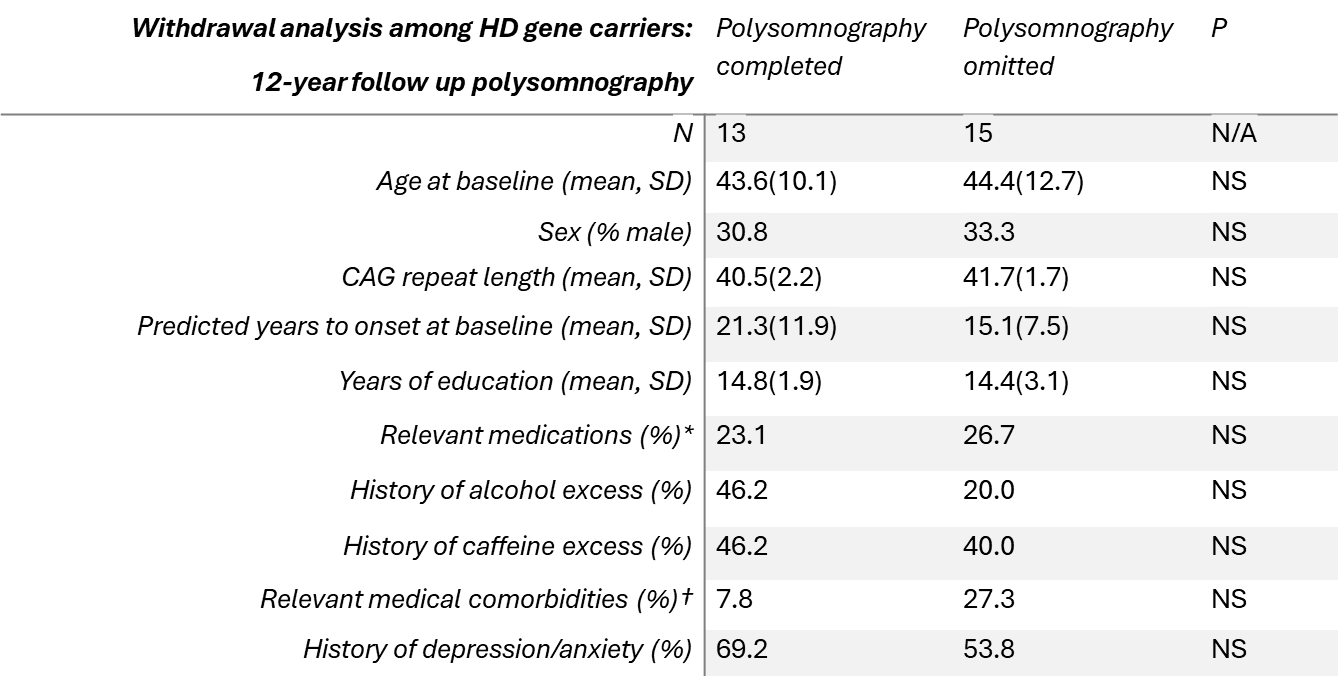
***

**Supplementary Table 6. Withdrawal bias analysis among HD gene carriers: polysomnography at 12-year follow up.** N/A=non-applicable. NS=non-significant. Group differences assessed by independent Student’s T-test or Chi square/Fisher’s exact test. Relevant medications in those who undertook polysomnography(PSG): carbamazepine(n=1), SSRI(n=2), beta blocker(n=1). Relevant medications in those who omitted PSG: SSRI(n=3), SNRI(n=1), benzodiazepine(n=1), statin(n=1), ACE inhibitor(n=1), calcium channel blocker(n=1). Relevant comorbidities in those who undertook PSG: post nasal drip(n=1). Relevant comorbidities in those who omitted PSG: perimenopausal symptoms(n=2), fibromyalgia(n=1) Where % and n are incongruent, this reflects isolated cases of missing data, polypharmacy and/or multiple comorbidities. Cohabitant status not recorded as no domestic recordings (actigraphy) undertaken at this timepoint.

| **HD gene carriers withdrawal analysis** | | **Remained in study to completion** | **Did not remain in study to completion** | **p** |
| --- | --- | --- | --- | --- |
|  | *n* | 23 | 5 | N/A |
| **Baseline cognitive** | *MOCA* | 27.3(1.7) | 28.0(1.6) | NS |
|  | *Trail A* | 31.4(10.6) | 30.8(8.5) | NS |
|  | *Trail B* | 52.6(20.1) | 59.0(25.3) | NS |
|  | *HVLT delayed* | 9.2(2.3) | 9.6(1.9) | NS |
|  | *SDMT* | 51.2(10.0) | 51.0(6.9) | NS |
| **Baseline affective** | *MADRS* | 4.7(7.2) | 6.4(10.1) | NS |
| **Baseline motor** | *UHDRS TMS* | 0.4(0.7) | 1.8(2.9) | NS |
|  | *UHDRS TFC* | 12.7(0.8) | 12.0(2.0) | NS |
|  | *UHDRS DCL* | 0.1(0.3) | 0.5(0.6) | NS |

**Supplementary Table 7. Withdrawal bias analysis among HD gene carriers across entire study: comparison of baseline clinical metrics.** N/A=non-applicable. NS=non-significant. Group differences assessed by independent Student’s T-test.

| **HD gene carriers withdrawal analysis** | | **Remained in study to completion** | **Did not remain in study to completion** | **p** |
| --- | --- | --- | --- | --- |
|  | *n* | 23 | 5 | N/A |
| **Baseline actigraphy** | *L5* | 1383(673) | 1503(678) | NS |
|  | *M10* | 26780(7642) | 24937(4489) | NS |
|  | *L5 onset* | 25.1(1.3) | 24.6(0.5) | NS |
|  | *M10 onset* | 33.2(1.5) | 33.8(2.2) | NS |
|  | *Relative amplitude* | 0.89(0.1) | 0.89(0.0) | NS |
|  | *Interdaily stability* | 0.52(0.1) | 0.53(0.1) | NS |
|  | *Intradaily variability* | 0.72(0.2) | 0.73(0.1) | NS |
| **Baseline PSG** | *TIB (hours)* | 8.56(0.8) | 8.58(0.7) | NS |
|  | *TST (hours)* | 7.2(1.2) | 7.1(1.2) | NS |
|  | *Sleep efficiency (%)* | 84.4(10.2) | 83.1(8.8) | NS |
|  | *Sleep onset latency (mins)* | 16.9(16.0) | 19.2(13.6) | NS |
|  | *Stage 1 (%)* | 10.2(4.3) | 12.6(6.0) | NS |
|  | *Stage 2 (%)* | 51.6(7.3) | 51.9(14.0) | NS |
|  | *Slow wave sleep (%)* | 18.2(8.3) | 14.3(7.1) | NS |
|  | *REM sleep (%)* | 20.9(4.2) | 21.2(8.5) | NS |
|  | *Wake after sleep onset (mins/hour)* | 6.3(3.9) | 6.9(3.8) | NS |
|  | *Awakenings/hour* | 1.3(0.6) | 1.4(0.8) | NS |
|  | *Limb movement arousals/hour* | 3.4(1.5) | 3.7(0.4) | NS |
|  | *Sleep stage changes/hour* | 26.3(8.5) | 26.1(2.1) | NS |

**Supplementary Table 8. Withdrawal bias analysis among HD gene carriers across entire study: comparison of baseline sleep metrics.** N/A=non-applicable. NS=non-significant. Group differences assessed by independent Student’s T-test.

***
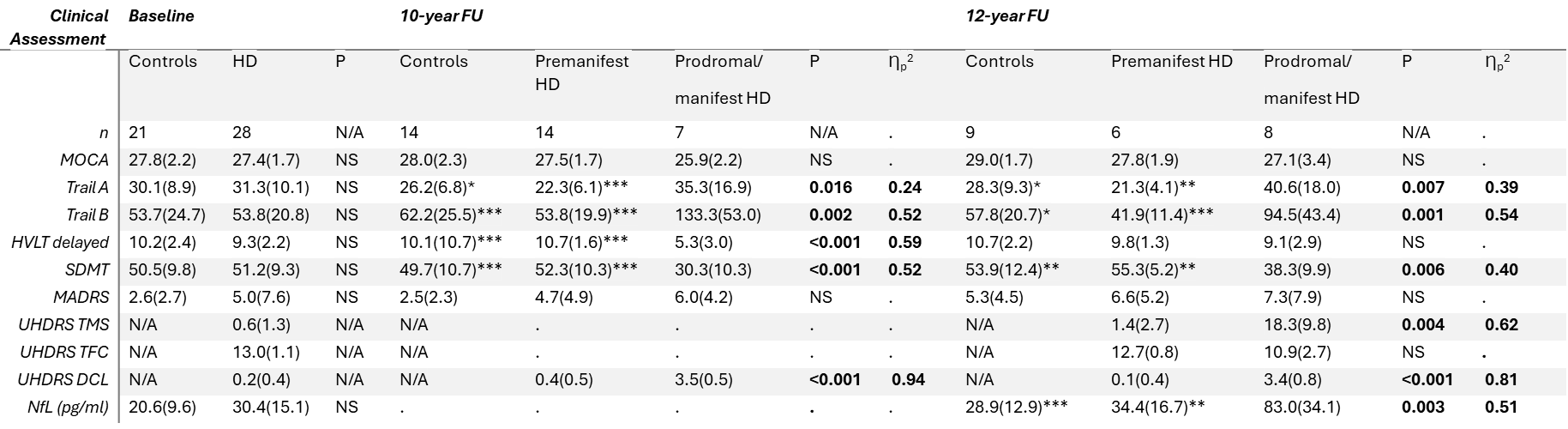
***

**Supplementary Table 9. Clinical assessment outcomes.** N/A=non-applicable. NS=non-significant. FU=follow up. Group differences assessed by ANCOVA adjusted for age, sex, CAG repeat length, MADRS depression score, relevant medication use and individual interval between baseline and follow up. Values represent totals or mean(SD) as applicable. *p<0.05, **p<0.01, ***p<0.001 in post hoc Tukey test versus prodromal/manifest HD group. Effect sizes reported where p<0.05 in both pairwise post hoc assessments vs prodromal/manifest HD. Note that direct comparison cannot be made between results at different timepoints, due to participant attrition between timepoints.

***
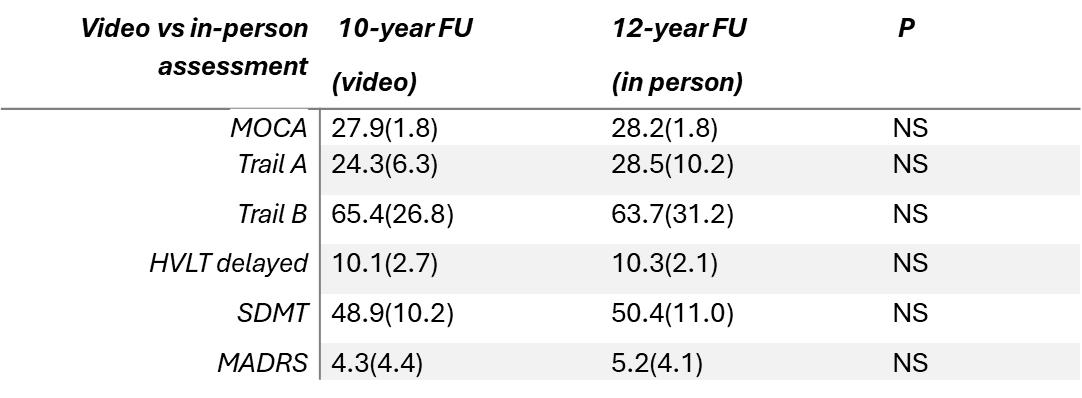
***

**Supplementary Table 10. Comparison of clinical assessments undertaken by video call versus in person.** Paired samples Student’s T-test applied to n=20 participants who undertook both forms of assessment. Values represent mean(SD). NS=non-significant. FU=follow up.

***
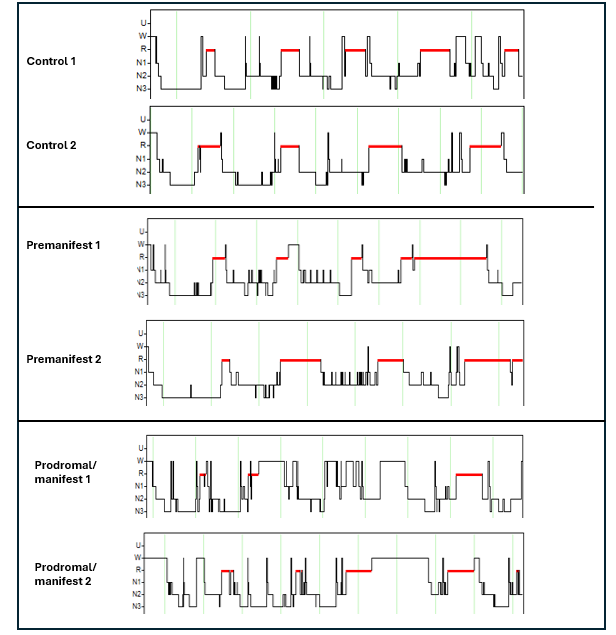
***

**Supplementary Figure 1. Example PSG hypnograms from six participants at 12-year follow up**, illustrating sleep stage instability and sleep maintenance insomnia in patients with prodromal/manifest HD. U=unscored, W=wake, R=REM (red), N1=stage 1 sleep, N2=stage 2 sleep, N3=slow wave sleep

***
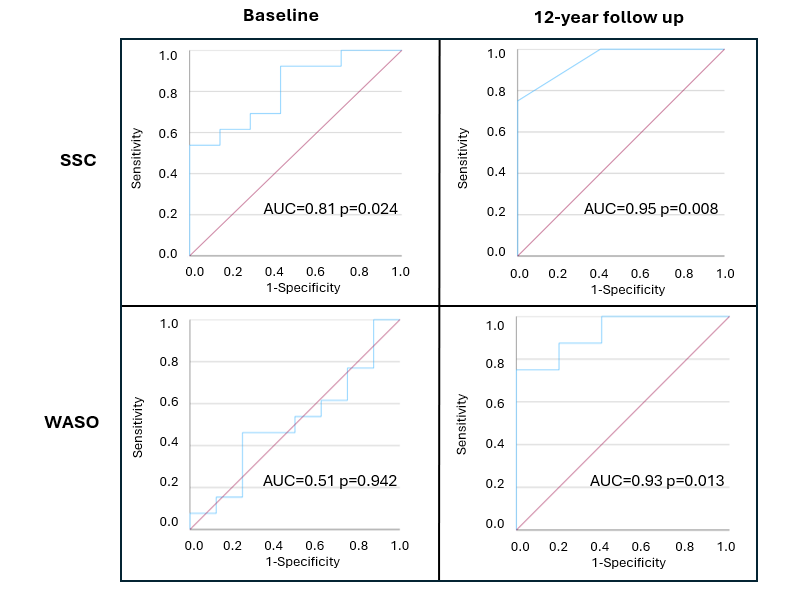
***

**Supplementary Figure 2. ROC curve analysis** of the ability of SSC and WASO to discriminate phenoconversion outcome among HD gene carriers at study completion, at baseline (left) and follow up (right). AUC= area under curve. N=21 at baseline; n=13 at follow up.
